# Multimodal Artificial Intelligence in Lung Cancer: From Data Integration to Precision Oncology

**DOI:** 10.3390/cancers18182953

**Published:** 2026-09-13

**Authors:** Turja Chakrabarti, Anthony G. Mansour, Xiwei Wu, Javier Arias-Romero, Isa Mambetsariev, Natalie Chang, Stephanie Delos Santos, Tamara Mirzapoiazova, Jeremy Fricke, Jae Kim, Michelle Afkhami, Chandana Lall, Ajaz M. Khan, Amanda Reyes, Matthew Lee, Debora S. Bruno, Colton Ladbury, Arya Amini, Ravi Salgia

**Affiliations:** 1 Department of Medical Oncology, City of Hope Comprehensive Cancer Center, Duarte, CA 91010, USAxwu@coh.org (X.W.); nachang@coh.org (N.C.);; 2 Department of Surgery, City of Hope National Medical Center, Duarte, CA 91010, USA; 3 Department of Pathology, City of Hope National Medical Center, Duarte, CA 91010, USA; 4 Department of Diagnostic Radiology, City of Hope National Medical Center, Duarte, CA 91010, USA; 5 Department of Medical Oncology and Therapeutics Research, City of Hope Cancer Center Chicago, Chicago, IL 60611, USA; 6 Department of Medical Oncology and Therapeutics Research, City of Hope Cancer Center Atlanta, Atlanta, GA 30265, USA; dbruno@coh.org; 7 Department of Radiation Oncology, City of Hope National Medical Center, Duarte, CA 91010, USA

**Keywords:** lung cancer, precision oncology, multimodal models, artificial intelligence, immunotherapy

## Abstract

Lung cancer remains the leading cause of cancer-related death globally, despite significant advances in diagnosis and treatment. Single biomarker approaches used clinically, such as programmed death ligand-1 (PD-L1) expression levels, have limited capacity for predicting treatment response. Multimodal data analysis using artificial intelligence (AI) offers an innovative scope to integrate diverse data sources—including radiologic imaging, digital pathology, genomics, immunohistochemistry, and Cell Painting morphology—to improve clinical predictions. This review aims to examine multimodal AI applications across the lung cancer treatment landscape related to such data sources. We analyze technical architectures spanning convolutional neural networks for imaging, vision transformers for pathology, and graph neural networks for genomics. We discuss how integrating and learning from heterogeneous data sources requires cross-attention fusion mechanisms. We further analyze critical studies demonstrating that multimodal AI clinical applications achieve superior predictive performance compared to unimodal biomarker methods. Multimodal AI models can augment clinicians in treatment selection, longitudinal monitoring using circulating tumor DNA (ctDNA), and variant interpretation through morphological profiling. We propose developing a multimodal AI model to optimize precision oncology for lung cancer.

## Simple Summary

Lung cancer remains the leading cause of cancer-related deaths. Non-small cell lung cancer accounts for most cases, many of whom are on immunotherapy. Current clinical practice relies on a single biomarker, like PD-L1 and tumor mutation burden, to make decisions on immunotherapy treatments. However, both biomarkers have reported inconsistent results in terms of predictive value for immunotherapy response and are thus not precise biomarkers. Therefore, a more robust method is needed to be able to accurately predict outcomes of interest in lung cancer, such as treatment response, treatment selection and diagnosis. Multimodal AI has the potential to revolutionize lung cancer treatment by integrating multiple different data types into a single framework to predict such outcomes. In this review, we examine current AI applications in lung cancer related to radiologic imaging, digital pathology, genomics, immunohistochemistry, and Cell Painting morphology and their potential to be incorporated into a proposed multimodal model.

## 1. Introduction

### 1.1. The Lung Cancer Challenge

Lung cancer emerges as the deadliest cancer worldwide, with approximately 1.8 million deaths annually, and accounts for nearly 18% of all cancer-related mortality [1,2]. In the United States alone, there were 218,893 newly reported diagnoses of lung cancer in 2022 [3,4]. Non-small cell lung cancer (NSCLC) accounts for approximately 85% of all lung cancer cases. Adenocarcinoma is the most common histological subtype, followed by squamous cell and large cell carcinoma [5]. Patients with advanced lung cancer have a poor prognosis, with stage IV disease demonstrating survival rates less than 10% at 5 years, despite advancements in diagnosis, screening, and treatment [6]. Challengingly, most patients present with locally advanced or metastatic lung cancer, which limits curative treatment options and often requires management with systemic therapy.

Treatment with immune checkpoint inhibitors (ICIs) targeting the programmed death ligand-1 (PD-L1) and PD-1 pathways has revolutionized the treatment paradigm for advanced NSCLC [7,8]. Selected patient cohorts have five-year survival rates approaching 25–30% when treated with ICIs, including pembrolizumab, atezolizumab, ipilimumab, and nivolumab [9,10,11]. This represents a significant survival advantage over chemotherapy alone. However, notable challenges persist in treatment selection as only 20–40% of patients achieve a durable clinical response from ICI treatment, while several experience disease progression and toxicity.

Current clinical practice predominantly relies on the PD-L1 tumor proportion score (TPS) assessed by immunohistochemistry (IHC) for immunotherapy selection. PD-L1 expression differs across NSCLC histologies, with squamous cell carcinomas showing higher rates of PD-L1 positivity than adenocarcinoma at some clinically relevant thresholds [12]. Considerable intertumoral and intratumoral heterogeneity nevertheless persists within histologic subtypes [13]. The KEYNOTE-024 trial established that pembrolizumab monotherapy is superior to platinum-based chemotherapy in patients with PD-L1 TPS ≥50%, leading to FDA approval and widespread clinical adoption [7]. However, this clinically used single biomarker approach demonstrates clinical limitations that have become increasingly apparent with expanded clinical experience. Not all patients with high PD-L1 expression respond to checkpoint inhibition, with objective response rates of 40 to 50% even in this biomarker-selected population [7,14]. Paradoxically, 15 to 20% of PD-L1-negative patients achieve meaningful and sometimes durable responses to immunotherapy, suggesting that PD-L1 expression incompletely captures the biological determinants of treatment benefit [15,16].

Tumor mutational burden (TMB), initially proposed as a complementary clinical biomarker-reflecting neoantigen load and immunogenicity, has shown inconsistent predictive value across clinical trials [11,17]. While the CheckMate-227 trial demonstrated improved outcomes with nivolumab plus ipilimumab in patients with high TMB, subsequent studies have not consistently validated this association, and TMB assessment remains non-standardized across platforms [18]. Co-mutations in genes such as STK11, KEAP1, and TP53 modulate immunotherapy response by altering the tumor microenvironment, yet these factors are not systematically incorporated into treatment algorithms [19,20]. These collective limitations reflect a fundamental gap wherein single-dimensional biomarkers inadequately capture the complex, multifactorial biology underlying treatment response in lung cancer.

### 1.2. The Multimodal Data Opportunity

A patient with lung cancer, during their treatment journey, generates a plethora of data that presents an opportunity for integrated analysis [21,22]. For instance, radiographic imaging provides comprehensive detail on tumor size, structural morphology, and positional information in relation to surrounding structures [23]. Accordingly, computational radiomics analyzes quantitative features regarding the tumor and surrounding microenvironment that may not be captured by human visual interpretation alone [24]. From a pathology perspective, digitized whole slide images (WSI) enable high-resolution characterization of tumor cell architecture and the spatial organization of immune populations, including tumor-infiltrating lymphocytes (TIL) [25,26]. Complementary multiplex tissue imaging can further resolve immune-cell populations such as tumor-associated macrophages (TAMs) and their spatial relationships within the tumor microenvironment [27,28]. Similarly, bioinformatic analysis of next-generation sequencing can reveal tumor mutational burden, actionable driver mutations, and novel genomic signatures [29]. IHC for PD-L1 can be used to measure the spatial distribution of checkpoint expression, while liquid biopsy with ctDNA allows for non-invasive dynamic molecular monitoring of tumor burden and mutational resistance patterns [30,31].

Traditionally, clinical workflows operate in siloes, with each specialty conducting analyses within the perspective of its discipline [21]. This results in a fragmented approach that fails to capture the synergy across data modalities. Tumor biology has distinct and complementary features: imaging reveals spatiality but not molecular characteristics; genomics identifies actionable molecular targets but cannot assess the immune landscape; pathology captures TIL dynamics but provides only a static snapshot; ctDNA enables dynamic monitoring but is limited in settings of low tumor burden [32]. Artificial intelligence and multimodal deep learning pipelines can combine diverse data sources through cross-attention mechanisms, creating complementary relationships between modalities that can overcome limitations and enable highly accurate predictive capabilities [21,22,33].

This review aims to provide an appraisal of multimodal AI applications in lung cancer. We examine the translational landscape encompassing data structures, technical architecture, and clinical implementation related to radiomics, digital pathology, genomics, IHC, ctDNA, and the emerging technique of Cell Painting morphological profiling. Additionally, we detail technical methods of data processing and modality fusion through deep learning pipelines. We review clinical applications of AI in the context of immunotherapy patient selection, optimal treatment duration, and therapeutic monitoring. We also consider clinical implementation challenges and pathways.

## 2. Methods

### 2.1. Operational Definitions

We refer to AI in the context of diagnostic methodologies, including radiomics, digital pathology, genomic profiling/molecular data, IHC, ctDNA, and Cell Painting; and its ability to enhance these methods’ diagnostic properties. We define “multimodal data integration” as input from at least two of these diagnostic methods, with “multimodal AI” integrating AI in the diagnostic process of at least two of these data sources. Conversely, we define “single-modality AI” as utilizing input from only one of these methodologies. However, some single-modality AI studies are included to establish the foundational increased benefit of implementing AI. These modalities can then be combined into a multimodal AI approach.

Multimodal data integration involves collection and integration of data from these numerous sources. Multimodal machine learning involves an AI model which can collect and process data from multiple sources and of various data types (i.e., images, molecular data, ctDNA information, etc.) Several multimodal AI fusion strategies are discussed: at the input-level (early fusion), feature-level (intermediate fusion), and decision-level (late fusion). Clinically validated multimodal AI have established increased accuracy when compared to diagnostic tests which exclude AI assistance.

### 2.2. Literature Search

During the literature search for this review, academic databases such as Google Scholar and PubMed were queried. Search terms included “non-small-cell lung cancer”, “multimodal AI”, and “emerging biomarkers”. References have been included from the past 30 years, though most were published within the last 10 years to ensure inclusion of recent technologies. Studies were selected that focused on implementation of uni-/multimodal AI models, discussions of their underlying mechanisms, input sources, and implementation of AI in oncology care.

## 3. Multimodal Data Sources in Lung Cancer

### 3.1. Medical Imaging and Radiomics

CT imaging is a core technology used for treatment planning and response assessment according to the Response Evaluation Criteria in Solid Tumors (RECIST) [34]. Radiomics involves quantitative analysis of features from CT images, including texture metrics derived from gray-level co-occurrence matrices and run length matrices, shape descriptors such as sphericity and compactness, and intensity statistics [23,24]. These radiomic features have demonstrated associations with molecular characteristics, including EGFR mutation status, histological subtype, and treatment response [35]. However, current standards in radiomics have limitations across different scanners and acquisition protocols, which require data harmonization methods [36]. Deep learning in radiomics represents a substantial advancement in three-dimensional convolutional networks (3D CNNs) to automatically learn hierarchical feature representations directly from volumetric CT data, with the potential to aid in tumor detection, classification, diagnosis and segmentation [37,38]. However, training deep learning models still requires expertly labeled training data, which is costly and time consuming, and thus represents a major bottleneck in the data curation step and warrants a shift towards unsupervised learning [37]. Technical architectures such as 3D-ResNet and 3D-DenseNet process entire tumor volumes, preserving spatial context across axial slices and capturing heterogeneity patterns that extend beyond the visible lesion boundary into the peritumoral microenvironment [39]. However, downsampling the volume via interpolation techniques comes at a cost of information loss [40]. Ardila and colleagues demonstrated an end-to-end deep learning model that achieved radiologist-equivalent performance in lung cancer screening on low-dose CT, establishing the technical feasibility of automated interpretation [41]. Deep learning architectures use techniques such as global pooling and heatmap generation to focus computational resources on diagnostically relevant regions, providing interpretability through spatial localization of disease patterns [42]. Machine learning also allows for the prediction of the invasiveness of non-solid, pure ground glass lung nodules [43]. Integration of Positron Emission Tomography (PET) imaging adds metabolic information regarding tumor hypoxia and glucose metabolism, providing complementary functional characterization [44]. Immune microenvironment features and molecular characteristics cannot be evaluated with high specificity by imaging alone, and thus require integration with tissue-based pathology and liquid biopsy modalities.

### 3.2. Digital Pathology and Histopathological Analysis

Digitized whole slide imaging has transformed pathology by generating gigapixel images with preserved tissue structure and cellular morphology. This digital revolution allows for computational analysis at a scale previously unattainable with traditional microscopy [26,45]. Coudray and colleagues developed deep learning models trained on H&E images that directly predicted molecular status of EGFR, KRAS and STK11 mutations from the histological slides without having to perform any molecular testing [46]. Limitations to more accurate predictions can be attributed to the small sample size of slides with gene mutations available for training use [46]. Nonetheless, this demonstrates how neural networks can detect morphological phenotypes that are imperceptible to human observation. These pathologic analyses have been replicated across multiple tumor types and extended to the prediction of survival outcomes in NSCLC [47,48]. The computational challenge of gigapixel WSI analysis has driven the development of specialized methods. Multiple instance learning (MIL) frameworks treat each slide as a set of patch-level instances, enabling slide-level predictions from weakly supervised annotations without exhaustive pixel-level labeling [49,50]. Vision transformers have demonstrated superior performance in pathology applications, with self-attention mechanisms capturing long-range spatial dependencies across tissue regions that convolutional approaches may miss [51,52]. Vision transformers demonstrate the potential of attention-based architectures to provide interpretability by highlighting regions that contribute to predictions, thereby facilitating applications such as quality assurance and pathologist review processes [51]. In addition, AI models extract prognostically relevant features, including TIL density and spatial distribution, tertiary lymphoid structures, and stromal characteristics that predict immunotherapy response of PD-L1 expression [53,54]. Analysis of PD-L1 spatial distribution patterns, including the interface between PD-L1-positive tumor cells and CD8+ T lymphocytes, may better predict checkpoint inhibitor efficacy than categorical TPS scoring [55].

### 3.3. Genomic Profiling and Molecular Data

Comprehensive genomic profiling through next-generation sequencing has become the standard of care for advanced NSCLC, with guidelines recommending broad molecular testing to identify actionable alterations [56]. Current targeted therapy options span multiple oncogenic drivers including EGFR mutations (present in 15–20% of Western and 40–50% of Asian populations), ALK rearrangements (3–7%), ROS1 fusions (1–2%), BRAF V600E mutations (2–4%), HER2 (ERBB2) mutations (3%), MET exon 14 skipping mutations (3–4%), RET fusions (1–2%), NTRK fusions (<1%), and KRAS G12C mutations (13%) [56,57,58]. Beyond individual driver mutations, NGS panels characterize TMB by assessing somatic mutation counts, copy number alterations, including amplifications and deletions, and mutational signatures [59].

Graph neural networks (GNNs) have been increasingly explored for integrating genomic data within multimodal AI systems [60]. By representing genes as nodes and known biological interactions as edges, GNNs propagate information across biological networks to generate patient-level embeddings that capture pathway-level alterations rather than isolated mutations [61,62]. This approach enables integration of mutation type, variant allele frequency (VAF), predicted functional impact, and co-mutation patterns within a biologically informed framework. However, amassing large labeled datasets to train the model can prove challenging, since reliably labeled data can be scarce in the bioinformatics field and access may be restricted due to privacy concerns [60]. In addition, these models have often been described as “black boxes” because the output of the model does not include an explanation for the different weights used internally to get to that output, making interpretation difficult [62]. The addition of Graph Layer-wise Relevance Propagation (GLRP), a post-processing step to explain GNN decisions and provide patient-specific molecular subnetworks, thus represents a useful attribution tool that could aid in therapeutic decisions [62]. Separately, tissue based genomic profiling offers data on a single time point, a key limitation in heterogenous solid tumors that can be resolved through integration with liquid biopsy methods [30].

### 3.4. Circulating Tumor DNA for Dynamic Monitoring

Liquid biopsy using ctDNA allows for a non-invasive exploration of the genomic composition of the tumor, while permitting serial monitoring over the treatment course [30,31]. Cancer cells release cell-free DNA into the bloodstream, which can then be detected by targeted sequencing or PCR- based approaches to identify tumor-specific mutations. Assessment of the VAF at baseline reliably quantifies the tumor burden and reveals prognostic insights independent of clinical stage [63]. Moreover, the timeline of ctDNA dynamics provides actionable clinical information as reductions in VAF or the clearance of detectable mutations can appear before radiographic response [31,64]. Bratman and colleagues demonstrated in a prospective study that ctDNA reduction after two cycles of pembrolizumab predicted progression-free survival (PFS) and overall survival (OS), establishing molecular response as a predictive biomarker for immunotherapy, thus demonstrating potential clinical use [30]. Likewise, early ctDNA reduction during durvalumab treatment correlated with improved clinical outcomes in lung and bladder cancer cohorts, as shown by Raja et al. [31]. Liquid biopsy can be utilized beyond response evaluation, as it enables the detection of resistance mutations, such as EGFR T790M or C797S, in patients on targeted therapies. As such, ctDNA guides clinical decision support before evidence of radiographic disease progression [65,66]. Serial ctDNA measurements yield temporal data patterns that are amenable to sequence modeling methods, such as long short-term memory (LSTM) networks and temporal transformers that model trajectories of treatment outcomes [67].

### 3.5. Cell Painting: Morphological Profiling for Functional Highlights

Cell painting is a novel high-content image-based assay that captures the morphology of cells by using multiplexed fluorescent staining. The standard protocol uses 6 fluorescent dyes to mark 8 cellular compartments: Hoechst 33342 for the nucleus; SYTO 14 for nucleoli and cytoplasmic RNA; concanavalin A for the endoplasmic reticulum; wheat germ agglutinin for the Golgi apparatus and plasma membrane; phalloidin for the actin cytoskeleton; and MitoTracker for mitochondria [68]. Automated image analysis derives over 1000 unique quantitative features from each individual cell, involving measurements of size, shape, intensity distributions, and texture patterns across organelles and cellular compartments. This morphological profiling renders functional readouts of cellular state, including responses to genetic changes and drug treatments, at single-cell resolution [69].

Tumor genomic sequencing often uncovers variants of unknown significance (VUS) that are specific to individual patients, with unknown functional and therapeutic implications. Cell-morphology-based variant impact phenotyping is emerging as a promising modality to address this fundamental clinical challenge [70]. Caicedo and colleagues generated deep-learning-based morphological signatures by profiling 375 lung cancer-associated gene perturbations (50 wild-type genes and 325 variant alleles) using Cell Painting methods. Notably, 83.2% of these variant alleles generated detectable phenotypic signals, illustrating that Cell Painting assays can support high-throughput assessment of variant impact [70]. Cell Painting alone is insufficient to convert a VUS into a clinically actionable alteration as formal reclassification requires multiple lines of evidence (i.e., guidelines, ancestry, bioinformatics, genome analysis, etc.), though it provides a strong source of data within the broader interpretation framework [71].

This method also showed specific morphological signatures of BRAF and KRAS oncogene variants and changes in tumor suppressors, providing the impetus for large-scale variant catalogs to direct the selection of precision therapy.

Model integration of Cell Painting with genomic and transcriptomic data provides complementary biological information. Way and colleagues profiled 1327 compounds across Cell Painting and L1000 gene expression platforms in A549 cells, demonstrating that the two modalities capture distinct aspects of cellular response [72]. Cell Painting showed greater reproducibility and phenotypic diversity, while the L1000 platform revealed more distinct molecular pathway signatures. Combined analysis improved mechanism-of-action prediction and gene-targeted identification compared with either modality alone [72]. The Joint Undertaking in Morphological Profiling (JUMP) consortium has generated an unprecedented public resource that, when complete, will capture Cell Painting data on 1.6 billion cells and their single-cell profiles, with paired chemical and genetic perturbations, enabling large-scale drug discovery and functional genomics applications [73]. Potential clinical applications in lung cancer include classification for treatment selection, patient-derived cell culture screens for personalized therapy, drug response prediction integrating morphology with genomic profiles, combination therapy optimization, and toxicity prediction through cellular health phenotypes.

## 4. Multimodal AI Architectures

### 4.1. Deep Learning Foundations and Modality-Specific Encoders

Multimodal AI systems employ specialized encoder architectures tailored to each data type’s unique characteristics (Table 1). This design creates fixed-dimensional embeddings suitable for downstream fusion [74]. For CT imaging, 3D CNNs such as 3D ResNet and 3D DenseNet process volumetric inputs (typically 128 by 128 by 128 voxels) through convolutional layers that preserve spatial relationships across all three dimensions [39,75]. Attention pooling mechanisms enable focus on tumor regions while suppressing background noise. Transfer learning from models pre-trained on large imaging datasets, including ImageNet and RadImageNet, accelerates convergence and improves generalization with limited oncology-specific training data [76]. The output from these encoders consists of 512-dimensional embeddings capturing tumor radiomic features.

Digital pathology encoders address the gigapixel scale of WSI by hierarchical processing, patch-level feature extraction, and the generation of embeddings for 224 by 224-pixel tiles, which are then aggregated through MIL frameworks to produce slide-level representations [50,51]. Foundation models trained on millions of pathology images—including GigaPath and Pathology Language and Image Pre-Training (PLIP)—provide powerful pre-trained encoders that transfer effectively to lung cancer applications with minimal fine-tuning [77,78,79,80]. Attention mechanisms at the aggregation layer learn which tissue regions most strongly influence predictions while providing interpretable attention maps highlighting diagnostically relevant areas. Genomic data encoders leverage biological knowledge via GNN architectures, with mutation matrices indicating the presence, type, and VAF of alterations serving as node features, while edges encode known gene–gene interactions [60,61]. Graph convolutional layers propagate information through the interaction network, with global pooling generating patient-level embeddings that capture pathway-level disruption patterns.

### 4.2. Multimodal Fusion Strategies

Fusion strategies are critical for integrating diverse data types and extracting synergistic information across modalities (Table 2) [81]. Early fusion approaches concatenate raw data or low-level features from multiple modalities before processing through shared neural network layers, allowing simultaneous processing of all available information but presenting challenges with disparate dimensionality, sensitivity to missing data, and limited ability to learn modality-specific representations before integration [82]. Alternatively, late fusion methodologies process each modality through independent networks to generate separate predictions, which are then combined using ensemble methods, voting schemes, or meta-learning approaches [83]. This approach has limitations: the absence of a single data type does not prevent overall prediction, but it may fail to capture complementary information from inter-modality interactions during feature learning.

Intermediate fusion with cross-attention mechanisms represents the current state of the art, enabling modalities to exchange information during representation learning rather than only at the input or output stages [21,22,33]. After modality-specific encoders generate embeddings, cross-attention layers compute attention weights between modality pairs, thereby enriching each representation with relevant information from other data sources. Transformer-based fusion extends this approach by treating modality embeddings as tokens processed by self-attention layers that learn complex inter-modal dependencies. Vanguri and colleagues demonstrated this architecture for immunotherapy response prediction through integration of radiology, pathology, and genomic data [21]. Attention weights provide interpretability by indicating the relative importance of each modality for individual patient predictions, supporting clinical adoption [84].

### 4.3. Training Strategies and Interpretability

Training multimodal AI systems presents unique challenges related to sample size, missing data, and class imbalance that require specialized strategies [85]. Transfer learning is a fundamental approach that uses pre-trained models on large datasets to initialize modality encoders, which are then fine-tuned on lung cancer cohorts. For pathology, foundation models pre-trained on millions of slides provide powerful starting points. For imaging, RadImageNet and similar medical imaging pre-training allow for optimized radiomic predictions. Missing data modalities represent a practical reality, as not all patients have all data types available. Training strategies to address this include modality dropout—randomly masking modalities during training to encourage robustness—learned imputation of missing embeddings, and collective approaches training separate models on modality subsets [86]. To prevent models from incorrectly predicting outcomes, weighted loss functions, focal losses, and synthetic oversampling are techniques required to account for class imbalance in oncology outcomes [87].

The stepwise nature of diagnostic medicine can also introduce significant spatiotemporal issues. Techniques such as prompt-based multimodal fusion, hybrid fusion, gated fusion, and tensor fusion can assist in alignment [88]. The proposed six-modality framework integrating, when available, radiomics, digital pathology, genomics, IHC, ctDNA, and Cell Painting, is complex and would require substantial training datasets as well as careful external validation to avoid overfitting.

For effective clinical use, interpretability methods are needed to transform “black box” models into user-friendly and comprehensible tools for clinical decision support [84,89]. An attention-weighted visualization is a powerful tool that provides clinicians with information on which data modalities or features have the highest impact on predictions, thus explaining a model’s analytical rationale. Gradient-weighted class activation mapping (Grad-CAM) identifies relevant regions in imaging and pathology data, enabling radiologists and pathologists to examine predicted focus areas [90]. SHAP (Shapley Additive exPlanations) Values measure and describe the importance of individual features [91]. Such understandable AI frameworks are critical for fostering physician trust, identifying potential points of failure, and satisfying regulatory requirements for clinical deployment. Enhancing the meaningful clinical application of AI infrastructure will involve a thorough study of model performance across varied multi-institutional cohorts, performance metrics with confidence intervals, and failure root-cause analysis.

### 4.4. Large Language Models in Precision Oncology

Foundational large language models (LLMs), which are primarily based on the Transformer architecture (instead of CNNs) have also been evaluated for their potential role in precision oncology [92]. Even without domain specific knowledge, these LLMs have been shown to perform well on medical exam questions from their pre-training on medical texts. A study by Vishwanath et al. found that general large language models outperformed specialized clinical tools like OpenEvidence on three separate medical benchmark tests [93]. The frontier models tested were OpenAI GPT-5.2, Google Gemini 3.1 Pro Preview and Anthropic Claude Opus 4.6 [93]. However, domain specific LLMs still show improved performed over general-purpose LLMs and adaption strategies include supervised fine-tuning, retrieval augmented generation, direct preference optimization, reasoning enhancement with chain of thought frameworks, and more [92]. Such models have shown high accuracy in diagnosis, classification, treatment and prognosis from real-world clinical tasks [92]. Furthermore, multimodal large language models show great potential for their ability to integrate multiple different data types and handle complex, cross discipline tasks. For example, TITAN, a vision-language foundation model for whole slide image pathology, has shown comparable performance to cutting edge pathology models like GigaPath [92]. Beyond standalone models, single-agent and multiagent AI systems have been proposed to aid in the complicated decision-making process in precision oncology. However, studies assessing performance on real-world clinical tasks remain limited and future analysis is required [92].

### 4.5. Potential Bias from Data Sources

Generalizability in multimodal AI models is a significant concern given the heterogeneity in data. For example, variability in radiomics, clinical practice, and other sources of input can all affect model performance. Transformer architecture has been shown to be robust against these differences [94].

## 5. Clinical Applications

### 5.1. Enhanced Diagnostic Accuracy

Multimodal AI tools can improve lung cancer diagnosis at multiple decision-making points during the patient’s clinical journey [37,46]. For example, integrating CT radiomic feature analysis with regression modeling of clinical characteristics (age, smoking history, family history, performance status, comorbidities) can help distinguish benign from malignant nodules. This may help avoid unnecessary invasive procedures, such as bronchoscopy and surgical biopsy [41,95]. In validation studies, deep learning methods have shown performance metrics equivalent to or better than those of radiologist interpretation (Table 3) [41]. Upon tissue acquisition, AI-augmented pathology can expedite histological subtyping and identification of rare histologies [46,50].

Developing multimodal AI tools for molecular subtyping without genomic sequencing is of particular interest. By integrating imaging and pathology features, models can accurately predict EGFR, ALK, KRAS, and other driver genomic alterations in validation cohorts (Table 3) [46,47,96]. This capability enables triage of patients for expedited molecular testing, provides provisional guidance when tissue is limited or testing infrastructure is unavailable, and offers a parallel prediction pathway while awaiting NGS results. Wang and colleagues demonstrated deep learning prediction of EGFR mutation status from CT imaging alone achieving an area under the curve (AUC) of 0.81 (95% CI, 0.79–0.83) in an independent validation cohort, indicating good discrimination between EGFR-mutant and wild-type tumors. Coudray and colleagues similarly demonstrated mutation prediction from H&E-stained histapathology images, with gene-specific AUCs ranging from 0.733 to 0.856 [46,96]. Integration across modalities further improves accuracy and reduces time to treatment initiation in resource-constrained settings.

### 5.2. Immunotherapy Patient Selection

A highly impactful clinical application of multimodal AI in lung cancer is the prediction of immunotherapy benefit, addressing the critical limitation of current single-biomarker PD-L1-based selection. The landmark study by Vanguri and colleagues at Memorial Sloan Kettering Cancer Center integrated radiology (CT-derived radiomic features), pathology (H&E-derived features, including TIL assessment), and genomics (mutation profiles and TMB) through a cross-attention multimodal architecture (Table 3) [21]. In a validation cohort of 385 patients with NSCLC treated with immune checkpoint blockade, the multimodal model achieved an AUC of 0.80 for predicting durable clinical benefit compared to an AUC of 0.65 with PD-L1 alone [21]. This represents a significant relative improvement when using multimodal AI compared to a single biomarker, with substantial clinical implications.

Independent validation studies have reproduced these performance gains across diverse healthcare settings and cohorts. As such, Sun and colleagues demonstrated that a machine-learning model which generated a radiomic signature assessing CD8+ T cell infiltration was able to predict immunotherapy response with an AUC of 0.76 across multiple cohorts [97]. Trebeschi and colleagues showed radiomic biomarkers generated from a machine-learning model predicting immunotherapy response with AUC of 0.72 to 0.76 in lung cancer and melanoma populations (Table 3) [98]. These multimodal approaches consistently outperform single biomarker strategies, distinguishing responders from non-responders of immunotherapy. Accordingly, multimodal AI could enable the accurate identification of high probability responders who may benefit from immunotherapy monotherapy, while predicted non-responders may be directed toward alternative treatments. Multimodal AI can also inform patients at increased risk of toxicity from immunotherapy-related adverse events [99].

### 5.3. Treatment Duration Optimization

Treatment duration decisions represent an emerging application of multimodal AI with significant clinical implications [9,100]. For patients achieving disease control on immunotherapy, there is no consensus on the optimal treatment duration. Current practice ranges from 2 years of fixed-duration therapy (based on clinical trial protocols) to indefinite continuation until progression or intolerable toxicity [9]. The CheckMate-153 Study compared continuous nivolumab with 1-year fixed-duration nivolumab, demonstrating improved PFS with continuation after 1 year but raising questions about whether selected patients could safely discontinue nivolumab [101]. Given annual ICI costs exceeding $150,000 per patient and the cumulative risk of immune-related adverse events, evidence-based duration optimization represents a substantial unmet need [100].

Multimodal AI enables the identification of patients who have achieved durable molecular and immunological responses, providing support for safe treatment discontinuation. Integration of imaging response depth and durability (distinguishing between complete response, deep partial response, and stable disease), ctDNA clearance indicating molecular complete response, and sustained immune signatures from tumor and blood, provides comprehensive response assessment [21,30,102]. Future applications include stratification of patients into risk categories following potential treatment cessation: low risk (<10% of 2-year progression probability), intermediate risk (10 to 30%), and high risk (>30%). Patients in low-risk categories may be candidates for treatment holidays or discontinuation with close surveillance. Longitudinal monitoring through ctDNA assays enables early detection of molecular relapse prior to radiographic or clinical relapse. Conversely, classification of patients as higher-risk may warrant continued treatment. Further studies are necessary to establish validity.

### 5.4. Real Time Monitoring and Adaptive Strategies

Early indication of treatment failure and real-time adaptation of therapies through multimodal AI, marks a transition from reactive to proactive oncology treatment decision-making (Table 3) [30,31,103]. Since serial ctDNA measurements can detect molecular progression weeks in advance of radiographic changes, a window of opportunity emerges for intervention before disease symptoms worsen [31]. Furthermore, inclusion of ctDNA dynamics with imaging data facilitates discrimination between true progression and pseudo-progression, a phenomenon which can be secondary to immunotherapy, in which apparent radiographic enlargement reflects tumor infiltration rather than tumor growth [104,105]. This difference has profound clinical significance, as pseudo-progression warrants continued treatment with the given regimen, whereas true progression compels a change in treatment.

Among patients undergoing targeted therapy, the identification of resistance mutations through ctDNA guides sequential treatment selection. For example, the detection of the EGFR T790M resistance mutation guides the transition from first or second-generation EGFR inhibitors to osimertinib [65,66]. Similarly, identification of MET amplification may indicate benefit from the addition of a MET inhibitor combination [106]. ctDNA provides clinical value from initial diagnosis to survivorship through identification of genetic alterations, monitoring of treatment efficacy, and prediction of relapse [107]. Limitations include low tumor burden, variable shedding, and clonal hematopoiesis [107,108]. However, multimodal AI models integrating ctDNA mutational dynamics, quantitative imaging changes, and clinical trajectory provide comprehensive resistance surveillance. Early prediction of treatment failure, at 6 to 8 weeks rather than the standard 12-week response assessment, enables a timely transition to alternative therapy, potentially improving outcomes while avoiding the toxicity and cost of ineffective treatment [31,103].

### 5.5. Demonstration of Clinical Applicability

While performance measures such as AUC do not necessarily translate to clinical applicability, multimodal approaches are well established in their improvement of predictive performance, as demonstrated in the studies referenced above. Multimodal AI predicts meaningful value over existing clinical and molecular decision-making approaches. It has also been shown to improve accuracy when predicting single biomarker efficacy, such as PD-L1. Determination of lung cancer treatment is multifactorial; however, single biomarkers such as PD-L1, are pertinent and often used in conjunction with driver mutations to select appropriate treatment options. Demonstration of multimodal AI as improving the accuracy of single biomarkers such as PD-L1 serves as foundational evidence for integrating multimodal AI throughout the patient journey. Further prospective studies are therefore warranted to evaluate the integration of multimodal AI as a clinical tool and its subsequent outcomes.

### 5.6. Limitations and Challenges of Multimodal AI in the Clinical Setting

Multimodal integration is constrained by the uneven availability of input data across patients. In advanced NSCLC, diagnosis often relies on small biopsies or cytology specimens from which material must be allocated among histologic assessment, PD-L1 IHC, and molecular testing [109]. Tissue exhaustion may therefore restrict acquisition of tissue-dependent modalities, while experimental approaches such as Cell Painting require specialized cell preparation, multiplex fluorescence staining and high-throughput imaging [68]. Models requiring a complete set of inputs may consequently exclude patients with missing modalities due to limited tissue or samples and introduce selection bias, particularly when data availability varies across institutions and clinical settings [109]. Clinically deployable frameworks should accommodate modality missingness and maintain reliable performance when only a subset of data streams is available [110].

## 6. AI Integration into Clinical Guidelines and Care Pathways

### 6.1. Current Guideline Frameworks

Clinical management of NSCLC is guided by evidence-based frameworks, including the National Comprehensive Cancer Care Network (NCCN) Guidelines, European Society for Medical Oncology (ESMO) Clinical Practice Guidelines, and the American Society of Clinical Oncology (ASCO) recommendations [111,112,113,114]. These guidelines provide algorithmic pathways for molecular testing, treatment selection, and response assessment. The current NCCN guidelines recommend broad molecular profiling for all patients with non-squamous NSCLC (with consideration for testing in squamous histology), along with PD-L1 IHC [111]. Treatment algorithms stratify patients based on molecular findings and PD-L1 expression to guide selection of targeted therapy, immunotherapy monotherapy, or chemo-immunotherapy combinations.

### 6.2. Opportunities for AI Integration

**Figure 1:**
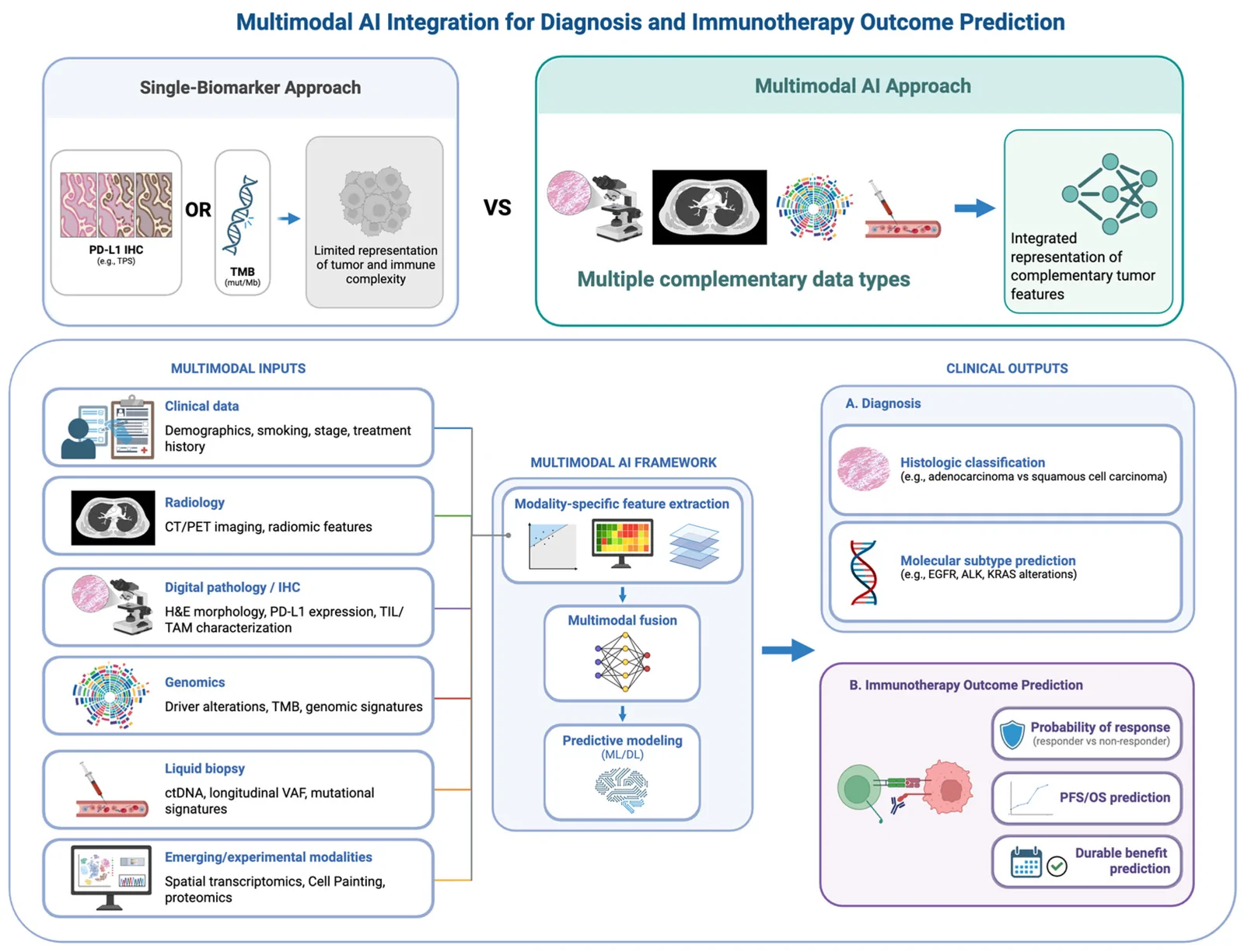
Multimodal AI framework for lung cancer diagnosis and immunotherapy outcome prediction. Made with BioRender.com.

Multimodal AI offers multiple integration points within established clinical pathways [111,112]. At diagnosis, AI-assisted nodule classification can support lung cancer screening programs by reducing false-positive rates and prioritizing high-risk patients for expedited workup [41]. During molecular workup, AI prediction of driver mutations from imaging and pathology can triage patients for reflex testing, provide provisional guidance when tissue is limited, and identify candidates for liquid biopsy when tissue-based testing fails [46,96]. For treatment selection, multimodal AI can augment PD-L1-based algorithms by integrating radiomic, pathologic, and genomic features to improve immunotherapy response prediction beyond single biomarker thresholds (Figure 1) [21,22].

Response assessment represents another key integration opportunity. Current guidelines recommend imaging-based response evaluation using RECIST 1.1 criteria at defined intervals [34]. AI-enhanced response assessment could integrate quantitative imaging features with ctDNA dynamics to provide earlier and more accurate response classification, distinguish pseudo-progression from true progression, and guide optimal timing for treatment decisions [30,31,35]. For treatment duration, where current guidelines lack consensus, AI-based risk stratification could inform evidence-based decisions about continuing or discontinuing immunotherapy. Additionally, AI has the potential to identify patients for clinical trial enrollment in real-time and improve accrual for precision oncology research [115].

### 6.3. Regulatory and Implementation Considerations

Integration of AI into clinical pathways requires navigation of regulatory frameworks governing clinical decision support software. The US Food and Drug Administration’s (FDA) Software as a Medical Device (SaMD) framework categorizes AI tools based on the significance of information provided and the healthcare situation [116]. AI tools intended to diagnose or drive treatment decisions for serious conditions require more rigorous premarket review than those providing supporting evidence. Regulatory review also differs for cemented algorithms versus adaptive or continuously changing algorithms where performance can be improved with additional data. To remedy this, the FDA finalized guidance in August 2025 on Predetermined Change Control Plans (PCCPs) which allows for future modifications regarding scope, methodology and validation to be made in a single submission rather than requiring a new submission per update [116]. This official new guidance by the FDA allows AI developers to pre-specify and get pre-approval for iterative AI model updates while maintaining regulatory compliance [116].

To be successfully incorporated into guidelines, the clinical efficacy of AI assisted pathways needs to be validated in prospective studies in comparison with standard of care algorithms. The NCCN, for example, has entered into a licensing agreement in November 2025 making NCCN Clinical Practice Guidelines in Oncology accessible through OpenEvidence’s AI-driven medical search platform, with all guideline content subject to NCCN’s existing evidence and quality control review [117]. This collaboration provides an AI-based interface for accessing established guideline content through OpenEvidence. Professional organizations such as the International Association for the Study of Lung Cancer (IASLC), have started to collect imaging data to establish consensus recommendations for the use of AI in thoracic oncology [118]. As more data becomes available from prospective trials and real-world evidence, validated AI models may emerge as valuable tools for molecular subtype prediction, response evaluation, and optimization of treatment duration.

## 7. Future Directions

The intersection of advances in deep learning techniques, the availability of large multimodal datasets, and the increasing clinical need for precision oncology creates a unique opportunity to develop the next-generation of AI-tools for lung cancer. Based upon the technical concepts and clinical applications reviewed in this article, we envision developing a multimodal AI platform that incorporates radiomics, digital pathology, genomics, IHC, liquid biopsies, and novel data types such as Cell Painting.

### Proposed Multimodal AI Architecture

**Figure 2:**
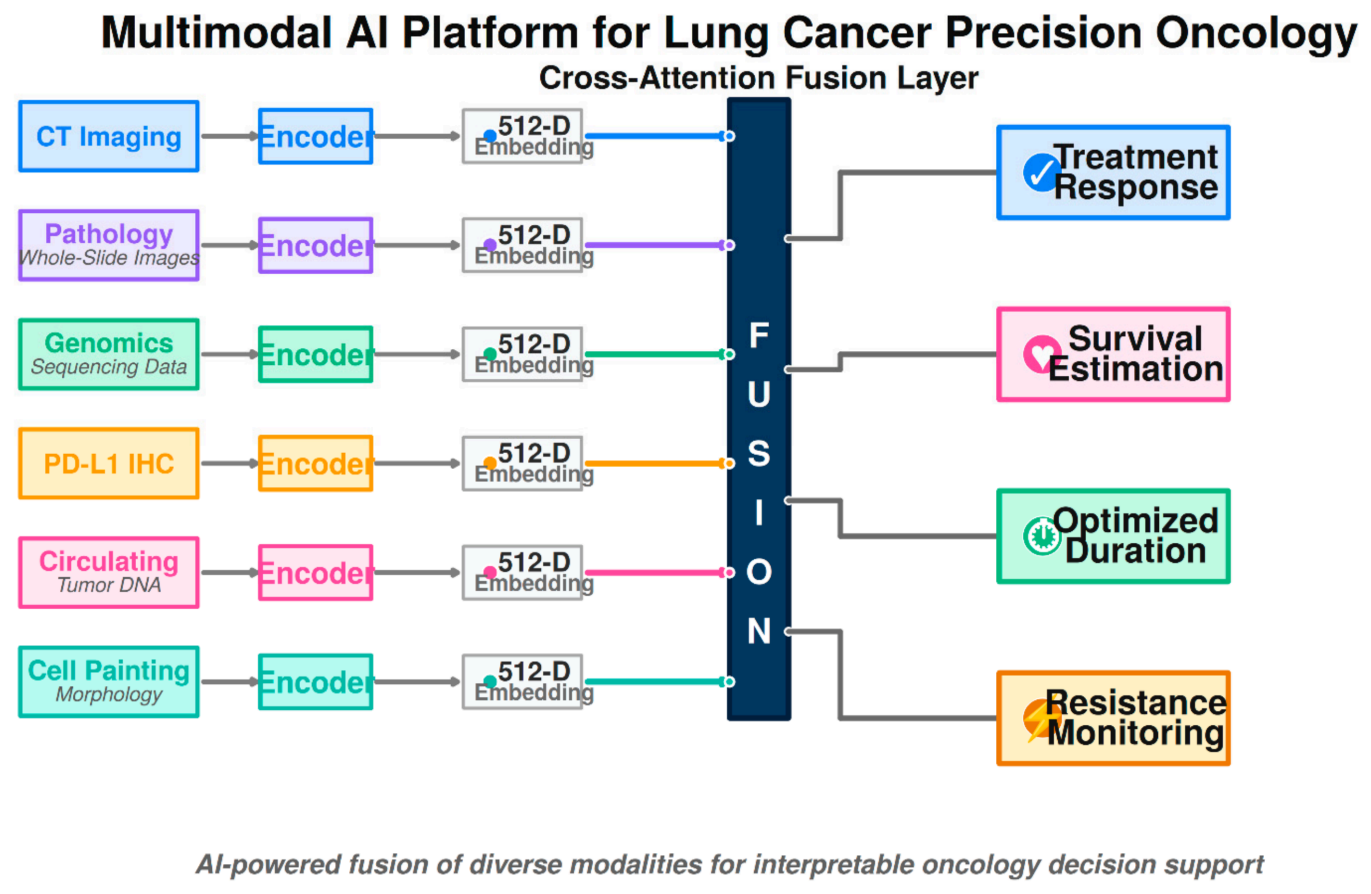
Schematic of the proposed multimodal AI platform for precision oncology in lung cancer. Our proposed hierarchical framework consists of 6 data modalities which are encoded into 512-dimensional vectors using modality-specific encoders. Inter-modality learning is facilitated via cross-attention mechanisms, resulting in clinically interpretable outputs for decision support.

The proposed platform will follow a hierarchical design with modality-specific encoders as the first layer of the technical architecture. These initial outputs will be fused through cross-attention mechanisms to generate integrated predictions for clinical decision support (Figure 2). This design overcomes the bottleneck of combining fundamentally different data types while retaining the rich and complementary information from each modality.

## 8. Modality-Specific Encoders

Each modality requires an encoder with an architecture adapted to the respective characteristics of the data. For radiomics, we will use a 3D ResNet that can directly process the volumetric data to extract features describing the tumor and the surrounding microenvironment. For digital pathology, we will utilize vision transformers with hierarchical attention that can process gigapixel WSI by learning features from patches that are aggregated across the slide by MIL [85]. To analyze genomic data, we will employ GNNs that model gene–gene interactions and allow for pathway level analysis. For IHC markers, we will use dense neural networks that model categorical and spatially resolved marker expression. Temporal transformer networks that model the time course of VAF measured longitudinally will be used for liquid biopsy analysis [119]. To leverage Cell Painting data, we will use CNNs that learn to describe cellular phenotypes from multiplex fluorescence images.

### 8.1. Cross-Attention Fusion Mechanism

The intermediate fusion layer will engage multi-head cross-attention, enabling each modality embedding to attend to all other modalities and learn complementary relationships [33,120]. This approach will allow dynamic weighting of modality contributions based on data availability and prediction context. The attention mechanism provides interpretability by revealing which modalities most strongly influence predictions for individual patients.

### 8.2. Clinical Decision Support Outputs

The platform will generate the following clinically actionable outputs: (1) treatment response probability scores for immunotherapy; (2) predicted survival outcomes (3) risk stratification to aid in treatment duration decision-making; (4) alerts for emerging resistance based on molecular and radiographic changes.

### 8.3. Development Roadmap

The clinical translation of multimodal AI models should follow four phases.

#### 8.3.1. Phase 1 Data Infrastructure

Automated extraction and harmonization of multimodal data from retrospective and prospective cohorts will be critical. Prospective cohorts should be as inclusive as possible and aim to have similar proportions of patients from various demographic backgrounds and clinical settings.

#### 8.3.2. Phase 2 Model Development

The AI model will be built to integrate diverse data inputs to allow for multimodal interpretation and meaningful clinical predictions. The use of foundation models and training techniques such as modality dropout, focal loss, and data augmentation, can help overcome challenges related to missing data points, class imbalance, and sample size.

#### 8.3.3. Phase 3 Validation

Validation of multimodal AI models across multiple independent clinical sites will be essential. Moreover, randomized clinical trials comparing AI-assisted novel biomarker-based therapy selection to standard of care approaches should be conducted. Progression-free survival and objective response rates should be the endpoints assessed in these trials.

#### 8.3.4. Phase 4 Deployment

Translation of the AI models into clinical practice can follow the FDA’s SaMD framework. For widespread clinical adoption, multimodal AI models will need to be integrated with electronic health records systems.

### 8.4. Emerging Technologies

Recent technological advancements that will aid in the development of multimodal AI systems include foundation models, federated learning, self-supervised learning, spatial transcriptomics, and AI driven biomarker and drug discovery. Foundation models that have been pre-trained on large-scale medical data can be used to perform transfer learning with disease-specific multimodal data cohorts that have relatively smaller sample sizes [121]. Federated learning approaches can be used to develop multimodal AI models by aggregating data across multiple sites without the need to share data, ensuring patient data privacy and increasing sample size. Self-supervised learning allows models to be trained on a much larger scale using unpaired multimodal data, largely bypassing the bottlenecked step of gathering paired multimodal data, which significantly improves performance [122]. Spatial transcriptomics provides gene expression data while preserving spatial information on tissue structure. Spatial gene expression may further facilitate comprehensive characterization of the TME. Integration of AI-based biomarker discovery with drug discovery has the potential to accelerate personalized treatment development, such as through identification of tumor-specific antigens for T-cell directed immunotherapies and personalized neoantigen vaccines [33,120,121,123].

### 8.5. Standardization and Clinical Translation of Multimodal AI

Multimodal AI could integrate complementary information across imaging, pathology, molecular profiling, and clinical data into a unified patient-level prediction. Expressing that prediction as a composite score could synthesize information otherwise distributed across separate biomarkers while providing a more interpretable output for risk stratification and clinical decision-making. Its meaning, however, would depend on a clearly defined clinical endpoint, treatment response, progression, survival, and toxicity represent distinct prediction tasks rather than components of a universal index [124]. A single value, however, may obscure discordance among modalities and provide limited insights into the contribution of individual inputs. Portability presents another challenge, where predictive performance and calibration may shift across populations and clinical settings, requiring evaluation in data representative of the intended use population [125]. Clinical use would ultimately depend on transparent reporting of the score’s inputs and rigorous external evaluation, including its discrimination, calibration and utility at clinically justified decision thresholds [124,125].

## 9. Conclusions

Multimodal AI has the potential to revolutionize the field of lung cancer precision oncology by providing a framework that integrates multiple types of clinical, imaging, pathological, and omics data into a robust model to predict outcomes of interest. We reviewed how multimodal AI systems have shown improved predictive power compared with single-biomarker-based methods. Specifically, integration of CT radiomics, digital pathology, and genomics has shown AUCs of 0.78–0.82 for predicting immunotherapy response compared with AUCs of 0.60–0.65 using PD-L1 expression alone [21,22]. In addition, the use of cross-attention fusion methods enables learning complementary information from multiple data types while providing explanations for predictions [33].

Moreover, multimodal AI can be used for a wide range of applications beyond treatment selection, such as predicting the optimal duration of therapies and monitoring resistance patterns in real time. For example, integration of imaging response depth, ctDNA clearance, and immune signatures can be used to identify patients who are at risk for disease progression. Accordingly, risk stratification can be used to guide decisions on whether to continue or discontinue immunotherapy [119]. Furthermore, incorporating AI analysis into Cell Painting has the potential to interpret VUS, thereby expanding the scope of actionable genetic changes.

Foreseeable implementation challenges such as data standardization, building computational infrastructure, and navigating regulatory issues will require coordinated multi-stakeholder efforts to overcome [85]. As data from prospective clinical trials and regulatory approvals become available, we envision that multimodal AI models will become a part of standard clinical practice. Ultimately, the long-term vision of precision oncology to personalize the selection, duration, and adaptation of therapies based on individual patient and tumor characteristics, will be invigorated by the use of multimodal AI.

## Figures and Tables

**Table 1:** Modality-specific deep learning encoder architectures.

Modality	Architecture	Input Dimensions	Output	Pre-Training Source	Reference
CT Imaging	3D-ResNet, 3D-DenseNet	128 × 128 × 128 voxels	512D embedding	RadImageNet, Med3D	[39,75,76]
Digital Pathology	ViT + MIL aggregation	224 × 224 patches → WSI	512D embedding	GigaPath, UNI, PLIP, CTransPath	[51,52,77,78,79,80]
Genomics	Graph Neural Networks	Mutation matrix + interaction edges	512D embedding	STRING, pathway databases	[60,61,62]
PD-L1/IHC	Dense Neural Networks	TPS, CPS, spatial features	512D embedding	—	[21]
ctDNA	LSTM, Temporal Transformer	Serial VAF timepoints	512D embedding	—	[67]
Cell Painting	CNN, ResNet	6-channel fluorescence images	512D embedding	JUMP consortium	[68,73]

Abbreviations: CNN, convolutional neural network; CPS, combined positive score; D, dimensional; LSTM, long short-term memory; MIL, multiple instance learning; TPS, tumor proportion score; VAF, variant allele frequency; ViT, Vision Transformer; WSI, whole-slide image.

**Table 2:** Comparison of multimodal fusion strategies.

Strategy	Mechanism	Advantages	Disadvantages	Reference
Early Fusion	Concatenation of raw data or low-level features before processing	Simple implementation; joint processing of all information	Dimensional heterogeneity; sensitive to missing data; limited modality-specific learning	Nigam et al. [82].
Late Fusion	Independent processing per modality; combination of predictions via ensemble or voting	Robust to missing modalities; allows specialized encoders	Fails to capture inter-modal synergies; misses complementary information	Peng et al. [83].
Intermediate Fusion	Cross-attention mechanism enabling modalities to exchange information during representation learning	Learns complementary relationships; dynamic modality weighting; interpretable attention maps	Higher computational cost; requires aligned training data	Vanguri et al.; Lipkova et al. [21,33].
Graph-Based Fusion	Patients as graphs with modalities as nodes; GNN propagation across modalities	Captures complex inter-modal relationships; flexible structure	Complex architecture; requires graph construction	Boehm et al. [22].

Abbreviations: GNN, graph neural network.

**Table 3:** Summary of clinically validated AI applications in lung cancer.

Clinical Application	Study	Data Modalities	AUC/HR	Comparator	Validation	Study Design	Validation Sample Size	AI Architecture	Advantages	Limitations
Molecular Subtyping	Wang et al. [96]	CT imaging (deep learning radiomics)	0.81	—	External	Retrospective, two center cohort, unimodal diagnostic accuracy study	241 patients	DenseNet based custom hybrid	Non-invasive genotype assessment, can use raw tumor image as input	Asian-only cohort, only EGFR tested
Molecular Subtyping	Coudray et al. [46]	H&E pathology (CNN)	0.73–0.86	—	Internal (TCGA)	Two cohort, unimodal classification and mutation prediction study	234 patients for normal vs. tumor classification, 181 patients used for subtype classification	Inception v3	High classification accuracy, ability to predict 6 of the 10 most commonly mutated genes in LUAD with AUC shown	Low tissue diversity, limited slide availability with positive mutation instances
ICI Response Prediction	Vanguri et al. [21]	CT radiomics + H&E pathology + genomics (cross-attention)	0.80	PD-L1: 0.65	Internal (n = 345)	Retrospective, single center, multimodal	247 patients with cross-fold validation, 98 patients for unimodal validation for radiology (n = 46) and pathology (n = 52) models	DyAM	Outperformed unimodal measures, handles missing data with masking	Restriction to single center, no external validation cohort, trained on RECIST endpoints instead of survival endpoints
ICI Response Prediction	Sun et al. [97]	CT radiomics (CD8+ T cell signature)	0.76	—	Multi-cohort	Retrospective, multi-cohort, elastic-net regression machine learning model	356 patients	---	Non-invasive, CD8 estimate from radiomic signature output also correlates with the abundance of other immune cells	Heterogeneous cohort, not all immune-phenotypes tested
ICI Response Prediction	Trebeschi et al. [98]	CT radiomics (lesion-level features)	0.72–0.76	—	External	Retrospective, multi-cohort, random forest machine learning model	70 patients	---	Non-invasive, wide availability of scans	Small subgroup sample size, prior heterogeneous treatment could have reduced accuracy
Nodule Classification	Ardila et al. [41]	LDCT (3D CNN, end-to-end)	0.94	Radiologists	External (Northwestern)	Retrospective, multi-cohort, risk prediction from low dose CT scans	907 patients	Custom CNN based on Mask R-CNN, 3D RetinaNet and 3D inflated Inception V1	Outperformed radiologist when prior scans were not available, no manual image segmentation needed	Operating points will be different in the clinic, lack of cancer outcome information available
Treatment Monitoring	Bratman et al. [30]	ctDNA dynamics (VAF reduction)	HR 0.29 *	—	Prospective	Prospective, five cohort, single institution clinical trial assessing ctDNA-based surveillance	94 patients	---	Non-invasive, potential predictive marker	Lack of early on-treatment ctDNA assessment
Treatment Monitoring	Raja et al. [31]	ctDNA early reduction (6 weeks)	HR 0.34 *	—	Multi-cohort	Exploratory biomarker analysis	101 patients	---	Non-invasive, reproducible, preceded radiographic change by months	Limited samples, some dVAF and imaging discordance

Abbreviations: AUC, area under the receiver operating characteristic curve; CNN, convolutional neural network; CT, computed tomography; ctDNA, circulating tumor DNA; H&E, hematoxylin and eosin; HR, hazard ratio; ICI, immune checkpoint inhibitor; LDCT, low-dose computed tomography; PD-L1, programmed death-ligand 1; TCGA, The Cancer Genome Atlas; VAF, variant allele frequency; LUAD, lung adenocarcinoma; DyAM, dynamic deep attention-based multiple-instance learning model with masking; RECIST, Response Evaluation Criteria in Solid Tumors; dVAF, change in mean variant allele frequency. Internal Validation is validation with data from the same source as the training data set; External Validation is validation with data from a different source than that of the training set and multi-cohort validation includes many different data sources. Prospective uses data from patients gathered during the study. * Hazard ratio for progression-free survival; lower values indicate improved outcomes with ctDNA reduction.

## Data Availability

No new data were created or analyzed in this study. Data sharing is not applicable to this article.
